# Six Susceptible-Infected-Susceptible Models on Scale-free Networks

**DOI:** 10.1038/srep22506

**Published:** 2016-03-03

**Authors:** Satoru Morita

**Affiliations:** 1 Department of Mathematical and Systems Engineering, Shizuoka University, Hamamatsu, 432-8561, Japan

## Abstract

Spreading phenomena are ubiquitous in nature and society. For example, disease and information spread over underlying social and information networks. It is well known that there is no threshold for spreading models on scale-free networks; this suggests that spread can occur on such networks, regardless of how low the contact rate may be. In this paper, I consider six models with different contact and propagation mechanisms, which include models studied so far, but are apt to be confused. To compare these six models, I analyze them by degree-based mean-field theory. I find that the result depends on the details of contact and propagation mechanism.

The development of communication technology and transportation has increased connectivity among people. Outbreaks of several new infectious diseases, such as AIDS, SARS, swine flu, and Ebola, have threatened human society. These diseases spread over networks of contacts between individuals. Computer viruses and worms spreading through the Internet have caused severe economic damages all over the world. Moreover, informations (for example, rumors, opinions, advertisement and innovations) spread through human networks. Understanding the intrinsic mechanism behind spreading phenomena in networks is an important and urgent task^1^,^2^,^3^,^4^,^5^.

Spreading phenomena, such as epidemic prevalence, have often been described by ordinary differential equations, that assume that the probability for an infected individual to encounter a susceptible host is uniform^6^,^7^. However, social networks are not uniformly mixed but are highly heterogeneous. Many social networks, such as telephone calls^8^, e-mails^9^, sexual relationships^10^, file actor collaboration^11^, citation networks^12^, and the Internet^13^, have scale-free properties. A network is called scale-free, if the distribution of degree (i.e., the number of links that connect to a node^14^,^15^,^16^,^17^) obeys a power law:


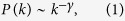


where *k* represents degree. For most real world networks, the exponent *γ* is between 2 and 3^14^,^15^. High degree nodes are called hubs. Spreading processes in such networks have been intensively studied recently^1^,^2^,^3^,^4^,^5^,^18^,^19^,^20^. It is well known that, for epidemic processes in scale-free networks, the high heterogeneity of connections leads to the absence of an outbreak threshold^18^,^19^. In this paper, six different types of contact and propagation dynamics on a network are considered to clarify the role of the hubs in general spreading phenomena on scale-free networks. Here, I use degree-based mean-field approximation^3^,^4^, where I assume that there are no correlations among the degrees of nearest neighbors. Since the purpose of this paper is to compare the six models in terms of the function of the hubs, this approximation is enough to explain the difference among them.

In this paper, I consider the susceptible-infected-susceptible (SIS) model as one of the simplest models for spreading phenomena. The SIS model is a model in epidemiology and is also known as the contact process model^6^. In an SIS model, a population with *N* individuals is categorize into two compartments: susceptible (*S*) and infected (*I*). The disease is transmitted only when a susceptible individual is in contact with an infected individual. When the case of information spreading is considered, *S* represents ignorant individuals and and *I* represents spreaders. The rate parameter *λ* is the average number per time unit of close enough contacts such that transmission can occur. By rescaling time, the recovery rate can be set to 1 without the loss of generality. In the case of a fully mixed population, the model is represented by two stochastic events:

1. At the rate of *λ*, the propagation event is performed. An individual is chosen at random. If the individual is infected, another individual is randomly chosen. If the second individual is susceptible, it becomes infected.
2. At the rate of 1, the recovery event is performed. Choose an individual at random. If the individual is infected, it recovers and becomes susceptible.

Mean field theory shows that the average density of infected individuals *ρ*(*t*) follows the rate equation


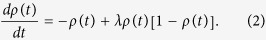


The equilibrium solution is calculated as


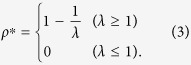


There is an outbreak threshold *λ*_*c*_ = 1, above which the prevalence can occur. Note that if the roles of susceptible and infected individuals are exchanged in the propagation event, result (3) does not change. In other words, the direction of the transmission is irrelevant to the spreading phenomena in a fully mixed population.

Here, I extend the model to include the network structure and the direction of the transmission. In the physics and mathematics literature, several definitions of SIS models on networks have been used. To study the differences, six types of SIS models on networks are considered. I assume that an individual is located at each node of a fixed network. The links of the network represent potential connections. It is assumed that an individual is activated at each time step. Then, the active individual can contact its nearest neighbors on the network. Two types of contacting mechanism are considered: (1) all neighbors or (2) only one neighbor is contacted at the same time. In addition, two possibilities for transmission are considered: an active individual is (a) the sender or (b) the receiver. I also consider the hybrid case (c): an active individual plays both roles. By combining the contacting and transmitting types, six models are constructed, as shown as follows (see also Fig. 1 and Table 1).

Although the definitions of Models 1a and 1b have been used as the standard definitions, they may be often confused. For example, in Pastor-Satorras and Vespignani’s papers^18^,^19^, an epidemic model was defined in the same way as Model 1b, but was analyzed as Model 1a. In another standard model, links rather than nodes are activated at random, and then the links transmit disease or information^1^. In degree-based mean-field approximation, this model coincides with Model 1a. On the other hand, although Model 2a has been studied both with theoretical analyses^21^ and numerical simulations^22^, Model 2b has been never as far as I know. The models with bidirectional transmissions (Models 1c and 2c) have been not considered so far. The purpose of this paper is to clarify the importance of the details of contact and propagation mechanism.

## Results

### Model 1a

In model 1a, if an infected individual is activated, all of its neighbors become infected. The propagation event in the previous section is replaced as follows

1. At the rate of 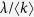, the propagation event is performed. Choose an individual at random. If it is infected, all of its neighbors get infected.

Here, the contact rate *λ* is divided by 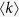 because the average number of contact per unit time is set to 1. Following the degree-based mean-field approximation^18^,^19^, consider the relative density *ρ*_*k*_(*t*) of infected individuals with degree *k*. The rate equation for *ρ*_*k*_(*t*) is given by


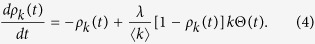


where the variable Θ(*t*) is the probability that a link transmits the disease. The first term of the right-hand side is the recovery event. The second term, which represents the propagation event, is proportional to the contact rate 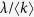, the density of susceptible individuals 1 − *ρ*_*k*_(*t*), the degree *k* and Θ(*t*). Since the probability that a link points to a node of degree *k* equals 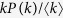, I obtain


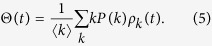


From Eq. (4), the equilibrium condition leads to


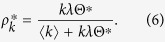


The total density of the infected in the equilibrium state is determined as


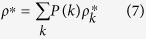


Substituting Eq. (6) into Eq. (5), I obtain a self consistent equation:


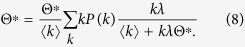


This self consistent equation always has a zero solution Θ^*^ = 0. Above the outbreak threshold, it has a nonzero solution. To calculate the outbreak threshold *λ*_*c*_, I consider the situation that the nonzero solution converges to 0. I divide the both sides of (8) by Θ^*^ and take the limit Θ^*^ → 0. As a result, I obtain


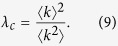


Since the second moment of the degree 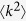 diverges for scale-free networks with *γ* ≤ 3, the outbreak threshold can vanish, i.e., *λ*_*c*_ = 0. Thus, disease can spread on scale-free networks no matter how low the contact rates may be. This result is essentially the same as the Pastor-Satorras and Vespignani’s analysis^18^,^19^.

### Model 1b

In Model 1b, I reverse the direction of the propagation process of Model 1a. An active susceptible individual gets infected if there is at least one infected neighbor. The propagation event is replaced as follows

1. At the rate of 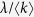, the propagation event is performed. Choose an individual at random. If it is susceptible and has at least one infected neighbor, it becomes infected.

In this case, the transmission probability in one time step is


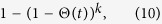


rather than *k*Θ(*t*). Therefore, the rate equation for *ρ*_*k*_(*t*) is rewritten as


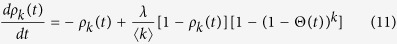


From Eq. (11), the equilibrium condition leads to


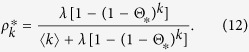


Substituting Eq. (12) into Eq. (5), I obtain a self consistent equation


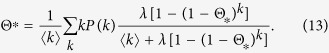


Considering the limit Θ^*^ → 0, it is obvious that the outbreak threshold is given by Eq. (9) once more. The absence of an outbreak threshold is seen as in Model 1a. Since 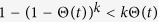, the equilibrium density ρ^*^ is smaller than that for Model 1a.

### Model 1c

Next, I consider a hybrid of Models 1a and 1b. Thus, the propagation event is described as follows:

1. At the rate of 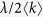, the propagation event is performed. Choose an individual at random. If it is infected, all of its neighbors becomes infected. If it is susceptible and has at least one infected neighbor, it becomes infected.

In this case, the rate equation is formed as a compound of Eqs (4) and (11):


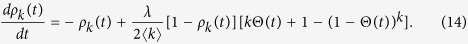


The equilibrium condition leads to


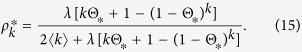


In the same way as for Model 1b, I obtain a self consistent equation


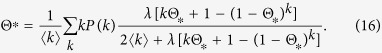


Thus, the outbreak threshold is given as Eq. (9). The equilibrium density ρ^*^ is intermediate between those for Models 1a,b.

### Model 2a

In the above three models, it was assumed that an individual contacts all of its neighbors simultaneously. Hereafter, I introduce three models to restrict the contacts to only one. In Model 2a, if an infected individual is activated, only one neighbor can become infected. The propagation event is replaced as follows.

1. At the rate of *λ*, the propagation event is performed. Choose an individual at random. If the individual is infected, choose another individual among its neighbors randomly. If the second individual is susceptible, it becomes infected.

In this case, the rate equation is rewritten as


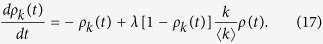


Here, instead of Θ(*t*) in Eq. (4) for model 1a, I need to use *ρ*(*t*), which is the probability that an individual is infected:


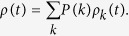


This comes from the fact that the number of transmissions provided by an infected individual is proportional to the degree of the activated individual in model 1a, while the degree of the activated individual is irrelevant to infectivity in Model 2a. Note that the multiplier *k* in eq. (17) represents the degree of neighbors of the active individual, rather than the degree of the active individual, in a similar way in eq. (4). The equilibrium condition leads to


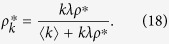


Substituting Eq. (18) into Eq. (7) and dividing both sides by ρ^*^, I obtain


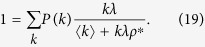


Taking the limit as ρ^*^ → 0, I find that the outbreak threshold is


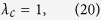


regardless of the degree distribution *P*(*k*).

### Model 2b

Here, I reverse the direction of the propagation process of Model 2a. An active susceptible individual contacts only one neighbor; if the neighbor is infected, the susceptible individual becomes infected. The propagation event is replaced as follows.

1. At the rate of *λ*, the propagation event is performed. Choose an individual at random. If the individual is susceptible, choose another individual among its neighbors randomly. If the second individual is infected, the first individual becomes infected.

In this case, the rate equation for *ρ*_*k*_(*t*) is written as


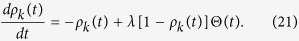


Here, 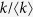 is removed in Eq. (4) for Model 1a. The equilibrium condition leads to


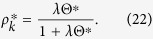


In this case, 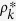 is independent of the degree, *k*. Thus, 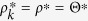. From eq. (22), the nonzero solution ρ^*^ > 0 satisfies


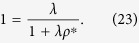


Thus, the analytical solution is obtained as


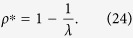


The density of infected individuals ρ^*^ coincides with SIS in a fully mixed population, regardless of the degree distribution. As a result, the outbreak threshold is given by


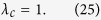


Jensen’s inequality leads to


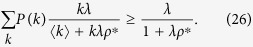


By comparing Eqs (19) and (23), I can deduce that in Model 2b, the equilibrium density ρ^*^ is larger than that in Model 2a.

### Model 2c

Finally, I consider a hybrid of Models 2a and 2b. Thus, the propagation event is given as follows

1. At the rate of *λ*/2, the propagation event is performed. Choose an individual at random and then choose another individual among its neighbors randomly. If the first individual is susceptible and the second one is infected, the first one becomes infected. If the first individual is infected and the second one is susceptible, the second one becomes infected.

In this case, the rate equation is formed as a compound of eqs (21) and (17):


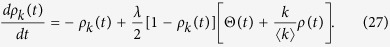


The equilibrium condition leads to


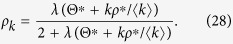


Substituting eq. (28) into eqs (5) and (7), I obtain


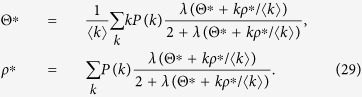


To estimate the outbreak threshold, I take the limit as Θ^*^ → 0 and ρ^*^ → 0:


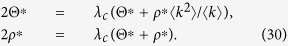


Solving Eq. (30), I obtain


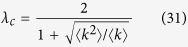


Although Eq. (31) is different from (9), for scale-free networks with exponent *γ* ≤ 3, the outbreak threshold vanishes, as in Models 1a, b, c.

## Discussion

**Figure 2:**
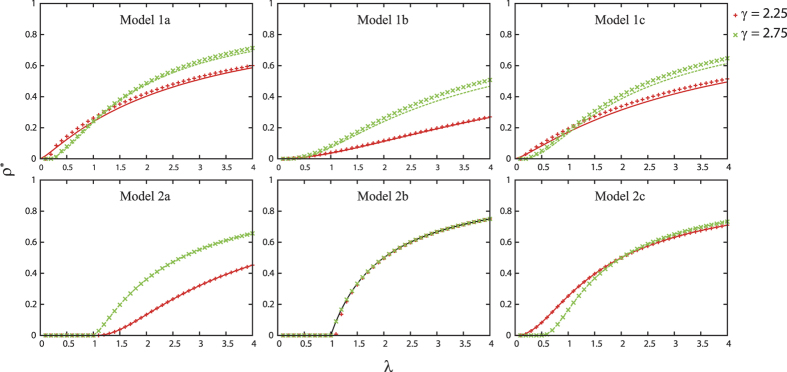
The density of infected individuals ρ* is plotted as a function of *λ* for the six different models, when *γ* is 2.25 (red) or 2.75 (green). The curves show the theoretical predictions, while the crosses represent the numerical results (see Methods section for the details of the calculations). In the numerical simulations, the system size is set to *N* = 100000 and each point is obtained by averaging over 10000 unit time after 10000 relaxation time on 10 different network realizations. Error bars are smaller than the size of the data point symbols.

I have analyzed the spreading phenomena on scale-free networks using six SIS models with different contact and propagation mechanisms. Figure 2 shows a decent match between theoretical predictions and numerical results. In Models 1a, b, c, the theory is not so accurate as in Models 2a, b, c. This is because the theory neglects the probability of an individual to reinfect the neighbor that had previously infected it^20^. This probability increases with the rate *λ*.

The theoretical predictions are summarized in Table 1. The six models were divided into two classes. In the first class, an active individual can contact all of its neighbors at the same time. Here, the outbreak threshold can vanish, regardless of the direction of propagation. In the case where active individuals are senders (Model 1a), the epidemic prevalence is larger than in the case where they are receivers (Model 1b). This is due to the fact that in Model 1b, propagation from more than one infected neighbors comes to nothing. In the second class, an active individual can contact only one neighbor. In contrast to the first class, when the active individuals are receivers (Model 2b), the epidemic prevalence is larger than that for Model 2a. This result may look counterintuitive at first sight; however, this result is not so surprising, because for Model 2b, an infected hub can transmit disease to more than one individual during a time step, in contrast to Model 2a. It is more surprising that in the case where the transmission is bidirectional (Model 2c), the outbreak threshold can vanish, while in the case the transmission is one way (Models 2a and 2b), the threshold remains finite. Thus, in the second class, the bidirectional transmission causes a qualitatively different effect. The intuitive explanation is that in Model 2c, a hub can play double role: a strong sender and strong receiver, as in models of the first class. However, it is emphasized that these are different types of phase transitions: 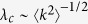 in Model 2c, while 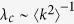 in first class.

In conclusion, the spreading phenomena depend entirely on the detailed mechanisms of contact and propagation. Thus, it is a matter there are more than a few works where it is not clear what mechanism is adapted. As the six models handle extreme situations, more complicated models have to considered to reproduce the practical situations. However, the result of this paper has a wide-range of applications in the study of the spreading phenomena, not only for epidemic diseases but also for such things as rumors and information. For example, in the case that an active individual receives information from one neighbor and sends it to another neighbor, rather than all neighbors, Model 2c indicates that the information can survive even if the transmission rate is very low. In real world networks, the links can be rewired adaptively depending on the state of their nodes. This paper is also expected to be a reference point when considering such adaptive networks.

## Methods

The numerical simulations are performed as follows. The degree distribution is given by


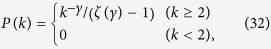


where *ζ*(*γ*) is Riemann zeta function defined as 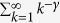. To generate uncorrelated simple graphs matching the input degree sequence, I use “degree.sequence.game” with method = “vl” in igraph/R package. The average degree 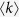 is 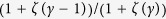 asymptotically, and decreases when *γ* increases. The initial condition is set to be random; two states have the equal probability (1/2).

The theoretical predictions in Fig. 2 are obtained in the following way. For Models 1a, b, c, I calculate numerically the nonzero roots Θ^*^ of the self consistent equations (8), (13) and (16), using the FindRoot package of Mathematica 10. Substituting the roots Θ^*^ into (6), (12) and (15), respectively, the degree distributions 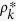 are obtained. From Eq. (7), I obtain the equilibrium density of infected individuals ρ^*^. For Model 2a, I calculate numerically the nonzero roots ρ^*^ of the self consistent equation (19). For Model 2b, I use Eq. (24). For Model 2c, I need to calculate numerically the the self consistent equation system (30).

## Additional Information

**How to cite this article**: Morita, S. Six Susceptible-lnfected-Susceptible Models on Scale-free Networks. *Sci. Rep*. **6**, 22506; doi: 10.1038/srep22506 (2016).

## Figures and Tables

**Figure 1:**
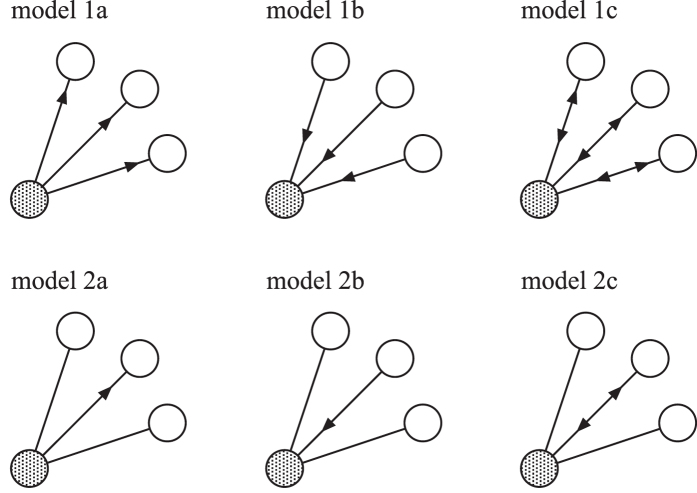
Schematic illustration of six models. Shaded circles represent activated individuals. Arrows mean the possibility of propagation. If the nodes to which the arrows point are susceptible and the source nodes are infected, then the propagation occurs.

**Table 1:** Properties of six models.

model	contacts	active individual	outbreak threshold	equilibrium density of infected
1a	all neighbors	sender	vanish	same as Pastor-Satorras and Vespignani^18^
1b	all neighbors	receiver	vanish	lower than Model 1a
1c	all neighbors	hybrid	vanish	intermediate of 1a and 1b
2a	one neighbor	sender	finite	lower than Model 2b
2b	one neighbor	receiver	finite	same as well-mixed case
2c	one neighbor	hybrid	vanish	
