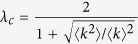# Corrigendum: Six Susceptible-Infected-Susceptible Models on Scale-free Networks

**DOI:** 10.1038/srep30862

**Published:** 2016-08-19

**Authors:** Satoru Morita

Scientific Reports 6: Article number: 2250610.1038/srep22506; published online: 03 03 2016; updated: 08 19 2016.

This Article contains errors in the following equations:

In Equation 30,


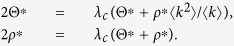


should read:


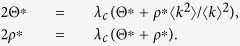


In Equation 31,


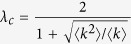


should read: